# Three-dimensional planning to optimize individual implant shape and location in thyroplasty surgery: How I do it

**DOI:** 10.1007/s00405-026-10434-2

**Published:** 2026-07-19

**Authors:** Annelies A. Mulder, J. E. Wachters, B. J. Merema, J. Kraeima, G. B. Halmos

**Affiliations:** 1 Department of Otorhinolaryngology – Head and Neck Surgery, University Medical Center Groningen, University of Groningen, Groningen, The Netherlands; 2 Department of Oral and Maxillofacial Surgery, University Medical Center Groningen, University of Groningen, Groningen, The Netherlands; 3 3D Lab, University Medical Center Groningen, University of Groningen, Groningen, The Netherlands

**Keywords:** Virtual Surgical Planning, Laryngeal framework surgery, Thyroplasty, Unilateral vocal fold paralysis, 3D printing

## Abstract

**Background:**

Although optimal voice outcomes in thyroplasty rely on precise targeting the affected vocal cord, the current surgical practice does not take the significant anatomical variations in this region into account.

**Method:**

A 3D virtual surgical planning is presented to complement the surgical technique with a patient-specific approach. This technique utilizes preoperative CT imaging of the thyroid cartilage to plan the optimal implant shape and target location. Patient specific 3D printed guides are used intraoperatively to guide the surgery for the optimal position of the implant.

**Conclusion:**

This paper describes a novel, patient-specific technique to enhance surgical precision in thyroplasty procedures.

## Introduction

Unilateral vocal fold paralysis (ULVP), is a common cause of dysphonia in the laryngology practice. A permanent solution can be provided by thyroplasty. The objective of this surgical intervention is to create a passive wall against which the healthy vocal cord can close. This is achieved by medializing the paralyzed side of the larynx by means of an implant. Optimal voice outcomes are contingent upon sufficient vocal cord closure. Therefore, the location of the implant needs to be precisely opposite to the unaffected vocal cord during phonation. Standard thyroplasty practice and implant systems do not, or only limited, consider interpatient anatomical variability [2]. However, anatomical variations of the thyroid and vocal cord are considerable [7; 8]. This threatens the objective of targeting the small target location of a 2–4 mm thick healthy vocal cord during phonation. Routinely made diagnostic CT scans for ULVP investigation contain precise information about individual vocal cord location [6]. We use this information in our novel, patient specific technique to determine thyroid window location and implant shape using 3D segmentation technique.

## Methods

### Standard thyroplasty practice

In our institution, we perform thyroplasty surgery according to the principles of thyroplasty Type I as reported by Isshiki [5]. He located the anterior commissure using external laryngeal landmarks. The trans-cartilaginous window, measuring on average 10 × 4 mm for women and 12 × 6 for men, is positioned 5-7mm posteriorly to the supposed location of the anterior commissure and parallel to the lower thyroid cartilage border.

Some general guidelines were also reported: The implant should not extend further medially than one-third of the vocal cord length to prevent over medialization [9]. Also sufficient posterior medialization is mentioned as key to success [3].

### Virtual surgical planning

Preoperatively, we virtually plan the procedure, utilizing a photon counting detector computed tomography (PCD-CT) scan of the thyroid cartilage during phonation, taking 0.4mm slices, a narrow field of view and a high resolution bone reconstruction algorithm. These settings allow to unambiguously visualize also non-ossified cartilage and the phonation ensures a clear imprint of the vocal cords in the vocal tract.

From the PCD-CT scan, a 3D model is generated of the entire thyroid cartilage and the vocal tract, using Mimics Core 27.0 software (Materialize, Leuven), by threshold-based segmentation, manually adjustments and validation by an experienced medical engineer.

**Fig. 1:**
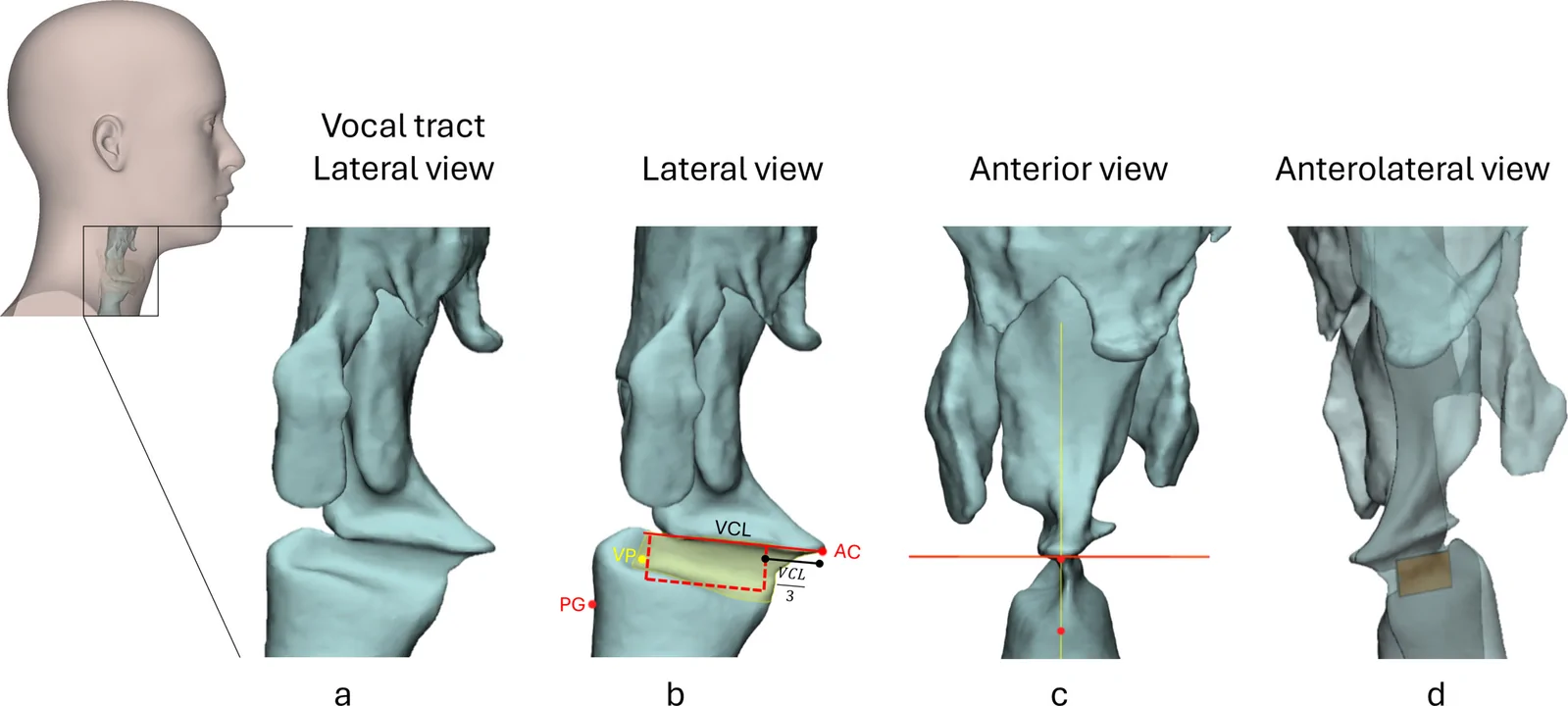
Implant target location identification on the vocal tract in phonation. On the lateral view of the healthy vocal fold imprint (**a**), the outline of the target location is drawn. For this, landmarks are identified: The Anterior Commissure (AC), the middle of the Posterior Glottis (PG) and Vocal Process (VP). The AC-VP distance, projected on the cranial surface of the vocal cord is the Vocal Cord Length (VCL) (**b**). The implant outline starts cranially just below the vocal cord’s cranial surface, anteriorly at one third of the VCL and ends posteriorly just before the VP, following the general guidelines. The implant thickness is similar to Isshiki’s description (b). The vocal tract midplane is defined by AC and PC and perpendicular to the healthy vocal cords cranial surface (**c**). The implant outline, projected to the vocal tract midplane is the target location of the implant (**d**)

Determination of the implant target location is explained in Fig. 1. The thyroid cartilage window is delineated following Isshiki’s guideline for window dimensions [5], but the long edges of the rectangular window are parallel to the direction of the vocal cord’s cranial surface.

**Fig. 2:**
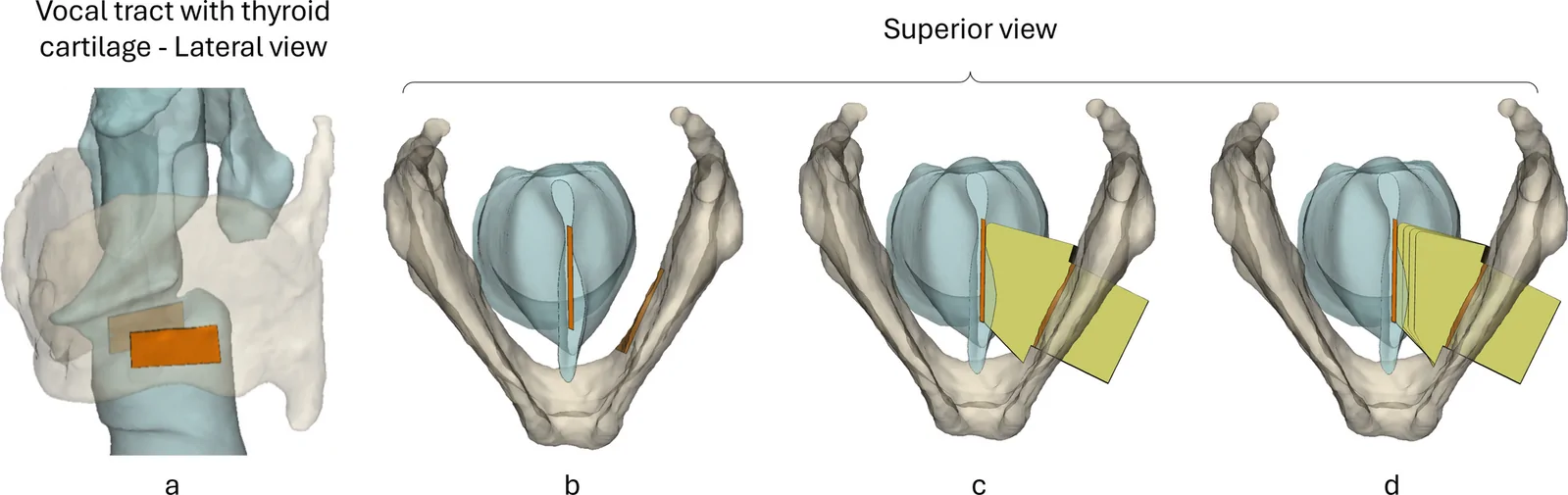
Implant construction. The target location at the midplane of the vocal tract, together with the window location on the thyroid cartilage (both in orange) (**a-b**) and an anterior and posterior 2 mm flange, define the implant main shape (**c**). 4 variations in implant lengths are made (**d**)

The implant shape is constructed based on the target location, the angle of the thyroid cartilage and the thyroid cartilage window, with a 2 mm flange anteriorly and posteriorly at the thyroid cartilage medial surface for stabilization of the implant (Fig. 2). To account for the unknown factor of soft tissue compression, 4 implant lengths are designed (Fig. 2d).

### Guide design

**Fig. 3:**
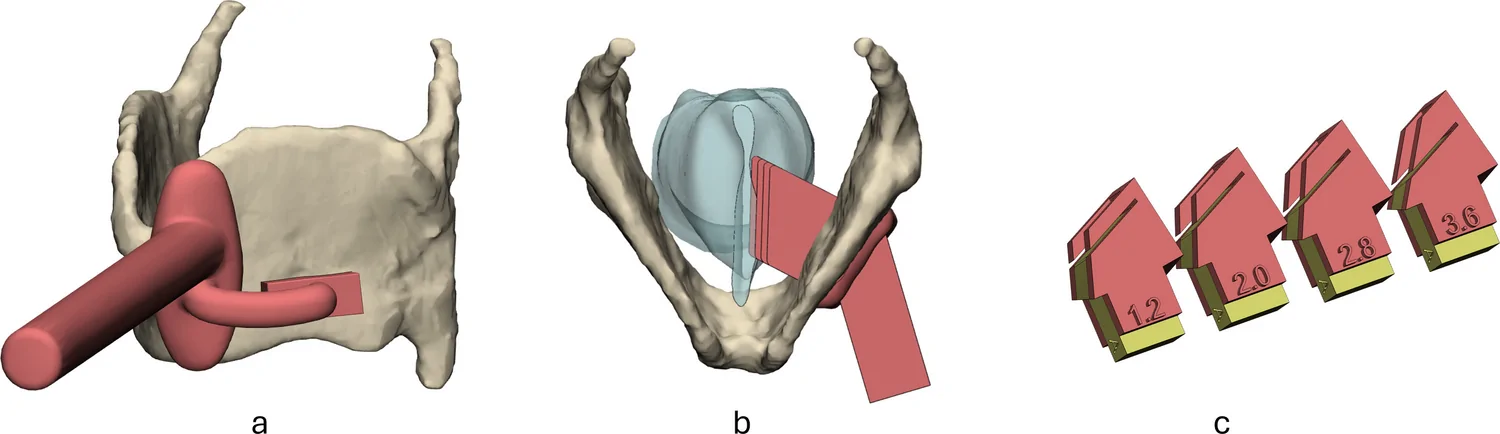
Guides to transfer the virtual plan to the surgical situation. A guide that fits the thyroid surface marks the window location (**a**). Fitting probes to test stem quality intraoperatively with various medialization lengths (**b**). Carving guides to obtain the planned implant shape (**c**)

To translate this virtual plan to the operating room, guides are designed and 3D printed in medical grade Polyamide-12. This includes a guide that fits to the thyroid cartilage surface and marks the planned window location. Furthermore, fitting probes with depths corresponding to the four implant lengths are provided. These are used intraoperatively to determine the clinically necessary amount of medialization, based on intraoperative phonation evaluation. Lastly, carving guides are made to carve the selected implant shape from an implantable silicone carving block (Fig. 3).

### Surgical technique

A patient is lying in a supine position on the operating table, slightly sedated with propofol. The anterolateral neck is disinfected and sterile draped, followed by local anesthesia lidocaine 2%, adrenalin 1:200.000.

**Fig. 4:**
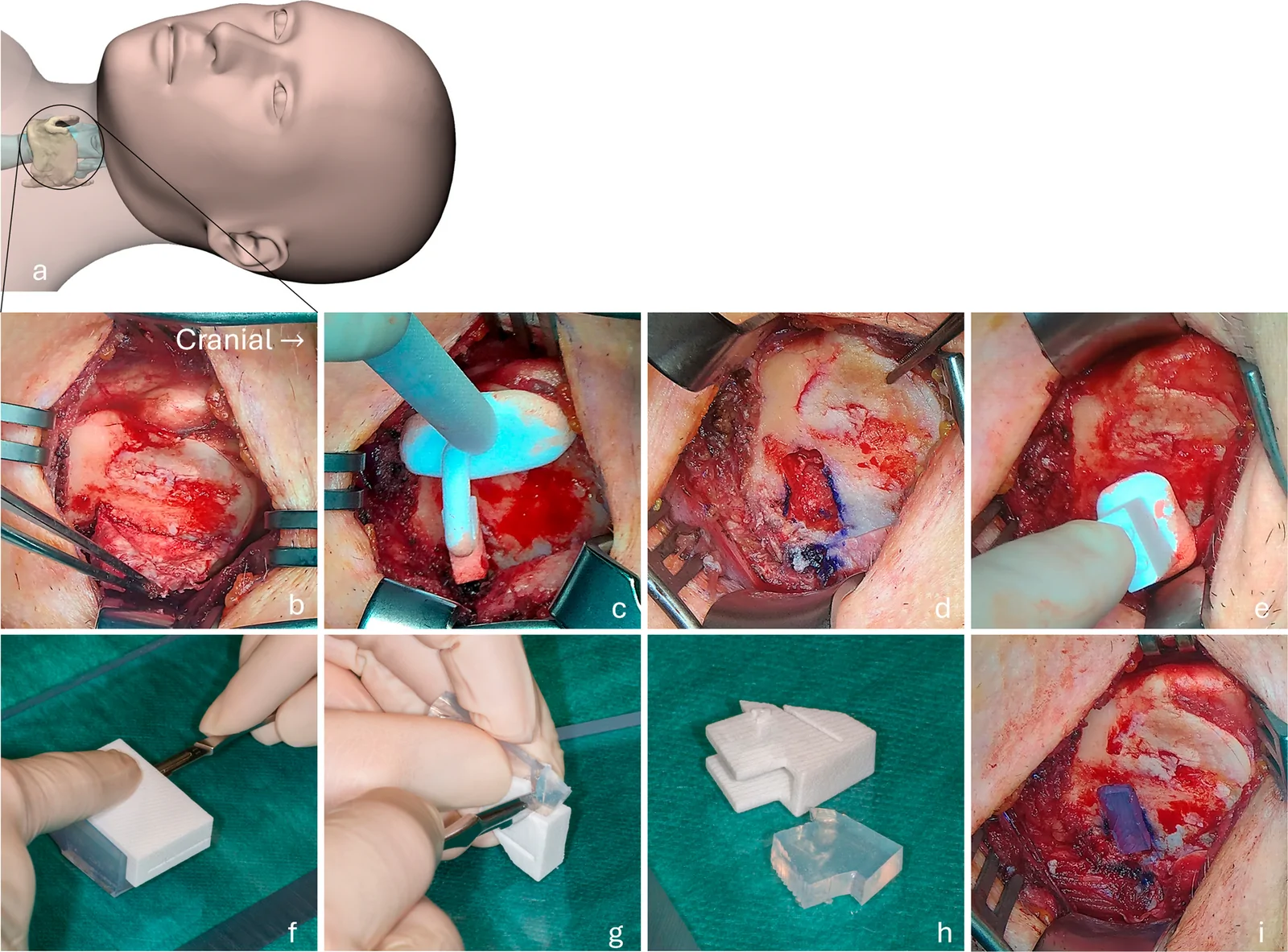
Surgical steps of thyroplasty surgery using 3D planning and 3D printed guides. **a**) Schematic model indicating photo context and orientation. **b-d**) Determining the window location and creating the window. **e**) Insertion of fitting probes to assess the patients voice result. **f-g**) Carving the implant according to the planned shape and measured depth. **h**) Carving guide with the resulting implant. **i**) Inserted implant in the thyroid cartilage

After horizontal skin incision on the supposed window level, the platysma and strap muscles are divided or retracted in order to expose the thyroid cartilage. The perichondrium is incised and shuffled aside. Using the personalized window guide and a surgical marker the window is marked on the ala, after which, using a no. 15 scalpel and a Lindeman drill, the window is opened and removed (Fig. 4b-d). With a Rosen’s knife the inner perichondrium is dissected.

The patient is asked to phonate while several fitting probes were subsequently tested under flexible laryngoscopic monitoring to decide on the final implant size (Fig. 4e).

Using a scalpel no. 11 and a carving guide corresponding with the fitting probe that led to the best voice quality, a silicone implant is crafted within 8 min (Fig. 4f-h), which is inserted into the trans-cartilaginous window (Fig. 4i). The wound is closed in multiple layers.

During the procedure 2 g cefazoline are administered, together with 20 mg dexamethasone.

## Discussion

Although thyroplasty is broadly recognized as a technically elegant procedure, many authors also acknowledge the need to take individual anatomical differences into account to perform more effective medialization [7, 8].

Our presented technique allows for tailoring the implant shape and location before surgery to the functional anatomy of the vocal tract, which is intraoperatively not visible. This offers an effective and precise way to achieve this customization. Although cost comparison of this method to commercially available systems is challenging due to differences in price between countries, we calculated that our method would cost over five times less than the Montgomery implant system in the Netherlands.

Various other studies have made methodological adjustments to the thyroplasty aiming to personalize the implant shape or location. Implant shape customization was done based on a 2D CT slice at the level of the vocal cords [1]. Also, an inflatable implant was developed to adjust the amount of medialization [4]. These methods do not consider possible angulated vocal cords with respect to the lower border of the thyroid cartilage [8]. Another method addresses this shortcoming by introducing a new instrument to adjust window location based on intra-operative testing, but no implant customization is considered, other than the standard sizes available in the Montgomery system [10]. However, these standard sizes are shown to be inadequate for female patients [2]. To our knowledge, the location of the healthy vocal cord has not previously been used as a starting point for adapting both the implant shape and the window location to this situation.

## Conclusion

This article outlines our novel technique to complement thyroplasty with a method to enhance precision. Further cases are needed to compare patient voice outcomes of this approach with the standard technique.

## Key points

- A 3D planning step before the thyroplasty procedure allows for tailoring implant shape and location to the individual patient
- No additional CT scan is needed, since a CT scan is routinely prescribed for ULVP patients
- Adjustments to the scanning protocol for ULVP patients may be needed:
- High resolution CT scanning is needed to visualize the entire thyroid cartilage
- Phonation during scanning is essential to visualize the healthy vocal cord imprint in the vocal tract, requiring careful timing.
- The implant's design ensures a symmetrical closure with the healthy vocal cord.
- Isshiki’s description regarding thyroid cartilage window dimensions is followed
- The healthy vocal cord determines thyroid cartilage window orientation
- The surgical procedure is only slightly adjusted by using 3D printed guides
- These guides make surgery faster and more precise, as they eliminate the need to conceptualize the lower border of the thyroid and determine the implant's shape in advance.
